# Inflammatory Stratification in Primary Sjögren’s Syndrome Reveals Novel Immune Cell Alterations in Patients’ Minor Salivary Glands

**DOI:** 10.3389/fimmu.2021.701581

**Published:** 2021-07-12

**Authors:** Tamandeep K. Bharaj, Lara A. Aqrawi, Siren Fromreide, Roland Jonsson, Johan G. Brun, Silke Appel, Kathrine Skarstein

**Affiliations:** ^1^ Gade Laboratory for Pathology, Department of Clinical Medicine, University of Bergen, Bergen, Norway; ^2^ Department of Health Sciences, Kristiania University College, Oslo, Norway; ^3^ Broegelmann Research Laboratory, Department of Clinical Science, University of Bergen, Bergen, Norway; ^4^ Department of Rheumatology, Haukeland University Hospital, Bergen, Norway; ^5^ Department of Pathology, Haukeland University Hospital, Bergen, Norway

**Keywords:** primary Sjögren’s syndrome, patient stratification, inflammatory severity index, minor salivary glands, adipose tissue, T cells, B cells, antigen-presenting cells

## Abstract

There is a critical need to deconvolute the heterogeneity displayed by the minor salivary glands of primary Sjögren’s syndrome (pSS) patients. This is challenging primarily because the disease etiology remains unknown. The hypothesis includes that initial events in the disease pathogenesis target the salivary glands, thereby triggering the development of focal infiltrates (≥50 mononuclear cells) and finally germinal center-like structures. However, the proportion of key mononuclear immune cells residing at these sites, in combination with the overall ratio of morphometric tissue atrophy and adipose infiltration within the minor salivary glands (MSG) parenchyma at distinct phases of inflammatory disease establishment and progression have not been quantified in detail. In this cross-sectional study, we intended to address this problem by stratifying 85 patients into mild (S1), moderate (S2), and severe (S3) stages using the Inflammatory severity index. We found that mild (<3%) and marked (≥3%) levels of atrophy were accompanied by the respective levels of adipose infiltration in the non-SS sicca controls (*p <*0.01), but not in pSS patients. The percentage of adipose infiltration significantly correlated with the age of patients (*r* = 0.458, *p <*0.0001) and controls (*r* = 0.515, *p <*0.0001). The CD4^+^ T helper cell incidence was reduced in the focal infiltrates of the MSG of S2 patients compared to S1 (*p <*0.01), and in S2 compared to S1 and S3 combined (*p <*0.05). CD20^+^ B cells increased from S1 to S3 (*p <*0.01) and S2 to S3 (*p <*0.01), meanwhile CD138^+^ plasma cells diminished in S3 patients compared to both S1 and S2 groups combined (*p <*0.01). The proportion of patients with anti-Ro/SSA^+^, anti-La/SSB^+^, and RF^+^ increased over the course of inflammatory disease progression and they were significantly more common in the S3 group relative to S1 (*p <*0.05). On the other hand, S2 patients measured a higher mean salivary flow relative to S1 and S3 patients combined (*p <*0.05). Our results demonstrate how the proposed Inflammatory severity index stratification revealed pathological cell and tissue-associated aberrations in the salivary component over the course of inflammatory progression, and their correlations to clinical outcomes. This could be directly transferred to the optimization of available diagnostic strategies applied for pSS patients.

## Introduction

Primary Sjögren’s syndrome (pSS) is a heterogenous autoimmune disease characterized by chronic inflammation that is manifested by mononuclear cell infiltration in the exocrine glands, namely the salivary and lacrimal glands. The minor salivary gland (MSG) tissue can be diagnostically examined by using the focus score (FS) which describes the number of cell infiltrates of at least 50 mononuclear cells within 4 mm^2^ (i.e., a focal infiltrate). Higher FS has been correlated with severe disease (1, 2) and associated with the occurrence of well-organized focal infiltrates closely resembling the germinal centers of secondary lymphoid organs with a light and dark zone (3). Due to their ectopic location in the salivary glands, such infiltrates are termed germinal-center like structures (GC), and have been reported in 25–30% of pSS patients (4, 5). The presence of GC in the diagnostic labial salivary gland have frequently been associated with antibody and immunoglobulin production (3) and a greater risk of lymphoma development (5, 6). Nevertheless, more studies are needed for a valid evaluation of their prognostic role (7), and hence, the GC^+^ biopsies of pSS patients do require special attention.

The heterogeneity in pSS also reflects on a histopathological level, where morphological features apart from the inflammatory lesions, such as salivary gland atrophy and adipose infiltration yet have not gained as much attention and diagnostic value (8). This could partially be ascribed to biological aging as has been found to correlate positively to the grade of atrophy or adipose tissue in the target organ of pSS patients for a long time (9–12). At the same time, pro-inflammatory mediators have been linked to atrophy and adipose infiltration in the glandular tissue (13, 14), and few studies have compared these respective tissue types morphologically by utilizing digital quantification techniques in a larger sample size.

Primarily, the focal infiltrations represent the hallmark feature in MSG biopsies of pSS patients, and these areas mainly comprise T and B lymphocytes and antigen-presenting cells (APCs). Nevertheless, the distribution of cell types within the focal infiltrates at distinct stages of inflammatory progression has been described by a few published studies (15), and a methodological concern lies in the patient stratification, where the GC^+^ and GC^-^ patients were not assessed separately.

Herein, we sought to investigate the immunopathological changes in the MSG of pSS patients by disseminating stages from the mild phase to highly developed lesions. Specifically, we aimed to create an Inflammatory severity index of disease activity to subgroup patients, in order to i) morphometrically quantify areas of atrophy and adipose infiltrations in the MSG of pSS patients and investigate a potential relationship between the two tissue types, and ii) measure the incidence and composition of key immune cell populations that comprise the focal infiltrates from mild to severe lesions. The infiltrating cell types analyzed included CD4^+^ T helper cells (Th cells), CD8^+^ T cytotoxic cells (Tc cells), FoxP3^+^ T regulatory cells (FoxP3 Tregs), CD74^+^ (class II MHC expressing) APCs, CD68^+^ macrophages, CD20^+^ B cells, and CD138^+^ plasma cells.

## Material and Methods

### Study Population and Design

This cross-sectional study included MSG biopsies from 85 pSS patients and 47 age-and gender-matched non-SS sicca tissue controls. As part of the diagnostic procedure the biopsies were obtained between the years 1989 and 2009 at the Department of Otorhinolaryngology, Haukeland University Hospital, Bergen. All patients fulfilled the American–European classification criteria (AECG) (16), and after a post-hoc re-classification 70 patients (80%) additionally fulfilled the revised 2016 ACR/EULAR criteria (17).

Medical records and subject charts were obtained from the Department of Rheumatology at Haukeland University Hospital and were retrospectively assessed for sicca manifestations, alongside laboratory and histopathological parameters. Sicca manifestations were tested using Schirmer’s test and sialometry, while serology findings included seropositivity for the following antibodies: antinuclear antibody (ANA), anti-Ro/SSA, anti-La/SSB, and rheumatoid factor (RF). Additional histopathological features included FS and GC in pSS patients at the time of diagnosis. Patients lacking biopsy data were excluded from the analysis. Moreover, since assessments of FS and GC are semi-quantitative methods where the histopathological tissue architecture might differ on multiple sections taken from the same gland, a re-evaluation of the FS and GC was conducted on MSG tissue from all pSS patients in order to eliminate potential discrepancies. This was performed on hematoxylin–eosin-stained sections in accordance with our previous laboratory examinations of such structures (18).

Patient stratification was carried out by stratifying the pSS patients into three distinct groups, where each group represented a disease stage according to the degree of inflammation in their MSG using the Inflammatory severity index. Patients with mild lesions (FS ≤1) were included in the first stage (S1), patients with moderate lesions (FS ≥2) were included in the second stage (S2), while patients displaying severe lesions and GC in the MSG tissue (FS ≥2 and GC^+^) were classified in the third stage (S3). Informed consent was obtained from all subjects included and the study was approved by the Regional committee of Ethics, Western-Norway (2009/686). The clinical and histopathological characteristics of pSS patients and non-SS controls are summarized in **Table 1**.

**Table 1:** Mean/median values and number (%) of all patients and non-SS control subjects in the total sample size positive for the clinical and histopathological characteristics.

	pSS patients				Statistical significance (*p*-value)			Non-SS sicca subjects
	Inflammatory severity index				Statistical comparison			
	Total, n = 85 (%)	S1, n = 44 (%)	S2, n = 24 (%)	S3, n = 17 (%)	S1 *vs*. S2	S2 *vs*. S3	S1 *vs*. S3	Total, n = 47 (%)
**Female**	82/85 (96.5)	42/44 (95.5)	24/24 (100)	16/17 (94.1)				43/47(91.5)
**Mean age (years)**	52.4 ± 1.22	52.8 ± 1.63	53.5 ± 2.51	49.9 ± 2.68				54.7 ± 1.41
ANA+^*^	52/79 (65.8)	25/41 (61.0)	14/23 (60.9)	13/15 (86.7)	0.993 ^a^	0.145 ^b^	0.106 ^b^	7/47(14.9)
Anti-Ro/SSA+^*^	47/85 (55.3)	20/44 (45.5)	14/24 (58.3)	13/17 (76.5)	0.310 ^a^	0.228 ^a^	0.029 ^a^	0/46 (0)
Anti-La/SSB+^*^	25/85 (29.4)	6/44 (13.6)	11/24 (45.8)	8/17 (47.1)	0.003 ^a^	0.938 ^a^	0.014 ^b^	0/46 (0)
RF+^*^	7/85 (8.2)	1/44 (2.27)	2/24 (8.33)	4/17 (23.5)	0.547 ^b^	0.212 ^b^	0.019 ^b^	2/47 (4.3)
Mean FS ^**^	2.14 ± 0.22	0.75 ± 0.06	3.21 ± 0.29	4.24 ± 0.66				0.21 ± 0.06
Median FS ^**^	1.00 [1.00–3.00]	1.00 [0.50–1.00]	3.00 [2.00–4.00]	3.00 [2.50–5.50]				0.00 [0.00–0.50]
**GC^+^**	17/85 (20.0)	0 (0)	0 (0)	17/17 (100)				0 (0)
Mean atrophy^***^	3.77 ± 0.26	3.56 ± 0.38	4.22 ± 0.51	3.68 ± 0.50	0.225 ^c^	0.653 ^c^	0.479 ^c^	4.33 ± 0.68
Median atrophy^***^	3.22 [2.08–5.15]	3.07 [1.80–4.86]						3.11 [1.50–6.82]
Marked atrophy***	47/85 (55.3)	23/44 (52.3)	14/24 (58.3)	10/17 (58.8)	0.632 ^a^	0.975 ^a^	0.645 ^a^	25/47 (53.2)
Mean adipose tissue^***^	8.82 ± 1.31	9.49 ± 1.80	8.92 ± 2.51	6.96 ± 3.06	0.949 ^c^	0.199 ^c^	0.145 ^c^	5.60 ± 1.35
Median adipose tissue^***^	2.81 [0.75–13.03]	3.52 [0.71–13.59]	3.72 [1.02–12.33]	1.36 [0.40–10.44]				1.78 [0.57–7.22]
Marked adipose tissue***	42/85 (49.4)	24/44 (54.5)	13/24 (54.2)	5/17 (29.4)	0.976 ^a^	0.116 ^a^	0.078 ^a^	19/47 (40.4)
Mean UWSF^****^	2.00 ± 0.27	1.70 ± 0.31	2.98 ± 0.62	1.31 ± 0.59	0.047 ^c^	0.012 ^c^	0.070 ^c^	1.48 ± 0.17
Median UWSF^****^	1.20 [0.45–2.35]	1.30 [0.30–2.00]	2.00 [1.00–4.00]	0.70 [0.00–1.20]				1.25 [0.85–1.80]
Decreased UWSF (hyposalivation)^*****^	45/77 (58.4)	24/42 (57.1)	10/22 (45.5)	11/13 (84.6)	0.373 ^a^	0.022 ^a^	0.102 ^b^	26/40 (65.0)
Mean Schirmer’s test^******^	13.37 ± 1.30	16.0 ± 1.94	10.1 ± 1.92	11.2 ± 2.90	0.067 ^c^	0.989 ^c^	0.143 ^c^	4.28 ± 0.66
Median Schirmer’s test^******^	10 [3.50–21.0]	11.25 [4.88–23.88]	7.25 [2.38–18.63]	5.50 [2.75–16]				3.00 [2.00–6.00]
Positive Schirmer’s test^*******^	37/85 (43.5)	14/44 (31.8)	13/24 (54.2)	10/17 (58.8)	0.072 ^a^	0.767 ^a^	0.053 ^a^	33/43 (76.7)

a Chi square test.

b Fischer’s exact test.

c Mann–Whitney U test.

Significant values (p <0.05) underlined.

S1, Mild stage; S2, Moderate stage; and S3, Severe stage along the Inflammatory severity index; ANA, Antinuclear antibody; RF, Rheumatoid arthritis; FS, focus score; GC, germinal center-like structures; UWSF, unstimulated whole saliva flow. Mean values presented with standard error of mean (SEM) when data was normally distributed, i.e. Shapiro–Wilk test p >0.05. Median [IQR] was used when the data followed a non-normal distribution.

*ANA, anti-Ro/SSA, and anti-La/SSB autoantibodies were detected using ELISA. RF was detected using Waaler’s test.

**A re-evaluation of the FS was carried out in all MSG. Values are the mean of the number of focal infiltrates/4 mm^2^ area comprising of at least 50 mononuclear cells. When FS <1 the numeric value was set to 0.5.

***The morphometric areas of atrophy and adipose tissue were quantified as a percentage of the total MSG parenchymal area, where ≥3% implicates marked levels, and <3% implicates mild levels (number of patients with mild levels not shown).

****Values are in ml/15 min. Eight subjects missing.

*****A salivary flow of ≤1.5 ml/15 min denotes a positive sialometry test.

******Values are in mm/5 min. Mean of lacrimal flow from both eyes.

*******A lacrimal flow of ≤ 5mm/5 min in at least one eye denotes a positive Schirmer’s test.

### Histomorphometric Analysis of Atrophy and Adipose Tissue

**Figure 1:**
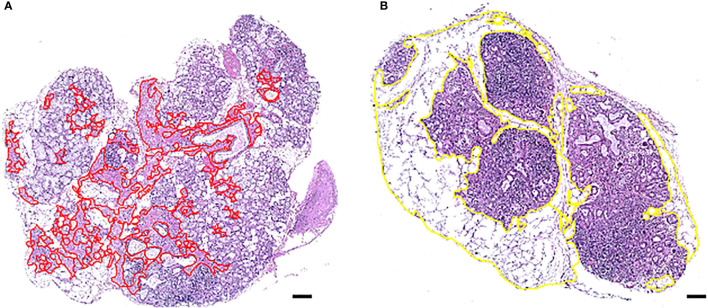
Morphometric distinction of atrophy and adipose tissue in one MSG from two patients with pSS. One representative salivary gland from each patient biopsy, using AperioImage Scope©, are shown. Atrophy and adipose tissue were quantified as a percentage of the total MSG parenchymal area. Parenchymal atrophy in the MSG biopsy of patient **(A)** was 9.1% and parenchymal adipose infiltration in patient **(B)** was 49,4%, where both possessed marked levels. Scale bar = 0.5 mm.

Hematoxylin–eosin-stained MSG sections were digitally scanned by the Hamamatsu NanoZoomer-XR digital whole slide scanner and converted into digital images available on Aperio ImageScope computer software (v12.4.3.5008; Leica Microsystems GmbH, Wetzlar, Germany). Atrophic and adipose areas within the parenchyma of the MSG were separately identified, morphometrically compartmentalized on the magnification of ×4 using the Pen tool in Aperio ImageScope (**Figure 1**), and finally divided by the total parenchymal area, giving the respective percentages of total atrophy and adipose tissue present in each biopsy. The MSG biopsies with mild (<3%) and marked levels (≥3%) of atrophy or adipose tissue were segregated due to a positively skewed distribution and to allow for a systemization of the MSG upon the grade of occupied tissues. All measurements were assessed blindly by two investigators (TKB, KS).

The analysis was conducted using four inclusion criteria. Firstly, structures located within atrophic or adipose regions that were maximum 3.5 µm² in size, such as horizontally cut capillaries or small ducts, were included in the measurements, while structures above 3.5 µm² were excluded. Other incompatible structures were also left out regardless of size. Secondly, only atrophy and adipose tissue inside the glandular parenchyma were measured. Thus, stromal tissue consisting of exterior fat, loose connective tissue, and other structures were not added in the calculation of the MSG area. Thirdly, the atrophic area comprised of damaged and/or irregular glandular tissue and densely packed connective tissue. Hence, loose connective tissue in the interlobular septa was not included, and focal infiltrates were left out as inflammatory cells covered the underlying tissue. Fourthly, adipose areas with at least three co-localized adipocytes were included in the analysis, whereas single and double adipocytes were excluded. Independent regions entirely or predominantly covered by adipose tissue were precluded from the analysis, unless they displayed structures attributable to MSG parenchyma, such as acinar cells or glandular ducts.

### Immunohistochemistry

#### Single Staining

Formalin-fixed paraffin embedded tissue of MSG from pSS patients were cut into 4 µm thick sections using a microtome (Leica Instruments GmbH, Nussloch, Germany). Next, the sections were placed on SuperFrost^®^ Plus microscope slides (Fisher Scientific, Waltham, MA, USA) and incubated overnight at 56°C. A total of 30 pSS patients from the histomorphometric analysis fulfilling the 2017 ACR/EULAR criteria (17) were enrolled in the analysis (**Supplementary Table 1**). MSG biopsies of patients with absence of focal infiltrates (FS = 0) were not included. As some of the biopsies from the current pSS patient cohort had been used in previous works (19, 20) biopsies with insufficient MSG tissue had to be excluded, especially considering the number of sections that had to be taken from each patient for the staining procedure. The single staining technique was performed using mouse-monoclonal anti‐human antibodies obtained from Agilent (Carpintera, CA, USA) that targeted CD4 (1:100 dilution, clone 4B12), CD8 (1:200 dilution, clone C8/144B), CD21 (1:100 dilution, clone 1F8), and CD68 (1:150 dilution, clone PG-M1). In addition, FoxP3 (1:100 dilution, clone 259D/C7, BD Biosciences Pharmingen, San Diego, CA, United States), and rabbit-polyclonal antibody detecting human CD74 molecule (1:250 dilution, Atlas Antibodies, Stockholm, Sweden) were included in the investigation.

The immunohistochemical analysis was carried out using the Envision FLEX+ kit (K8012, Agilent, Carpintera, CA, USA). After overnight incubation, sections were deparaffinized in xylene and rehydrated by serial dilutions of ethanol in distilled water (100, 96, and 70%). They were subjected to heat-induced epitope retrieval (HIER) in a microwave for 25 min and blocked with peroxidase for 5 min, followed by a 1:10 dilution of Normal goat serum (X0907, Agilent, Carpintera, CA, USA) in 3% Bovine serum albumin (BSA) solution for 20 min. Next, the serum was removed, and the sections were incubated with the primary antibody mixed in diluent (K8006, Agilent, Carpinteria, CA, USA) for 60 min, following EnVision FLEX+ horseradish peroxidase (HRP)—conjugated secondary antibody for 20 min. 3,3′-diaminobenzidine (DAB, K3468, Agilent, Carpinteria, CA, USA) was applied on the sections and excessive chromogen was washed away with distilled water after 10 min. The sections were then counterstained with hematoxylin (S3301, Agilent, Carpinteria, CA, USA) for another 10 min, dehydrated *via* a graded ethanol series, and finally mounted with Pertex (Histolab Products AB, Gothenburg, Sweden). Following epitope-retrieval, all incubations were performed at room temperature, and Tris-buffered saline (TBS) pH 7.6 was used as a washing buffer between all steps. For antibodies CD4, CD8, and FoxP3 normal goat serum was replaced by a serum-free Protein block (X0909, Agilent, Carpintera, CA, USA), and an additional amplification step where EnVision FLEX+ Mouse Linker was incubated for 15 min following the primary antibody was added. Normal goat serum was not used for the CD21 antibody.

#### Double Staining of CD138 and CD20

Double-staining with mouse-monoclonal antibodies targeting human CD138 (1:200 dilution, clone MI15, Agilent, Carpintera, CA, USA) and CD20 (1:3,000 dilution, clone L26, Agilent, Carpintera, CA, USA) was performed. Here, CD138 was the first primary antibody incubated for 60 min at room temperature and developed using DAB similarly to the single-staining-technique described above. The sections were then treated with Dual Endogenous Enzyme Block (S2003, Agilent, Carpintera, CA, USA) for 5 min, and incubated with the second primary antibody CD20 overnight at 4°C. The following day, sections were stained with Vulcan Fast Red Chromogen (Biocare Medical, Concord, CA, USA). The sections were counterstained with hematoxylin for 10 min, dehydrated, and mounted as described above.

#### Evaluation of Staining

In order to evaluate the staining in a replicable manner, the MSG sections were converted into digital images accessible on Aperio ImageScope software as described above. Staining of CD21^+^ follicular dendritic cells (fDC) was carried out in order to verify the presence of CD21^+^ networks centrally within the GC in the tissue and ensure accurate S3 patient stratification with regard to the Inflammatory severity index. The rest of the antibodies were included in the staining analysis to assess the percentages of each immune cell population within the focal infiltrates of MSG tissue. Focal infiltrates with minimum 50 mononuclear cells located periductally or perivascularly, in close proximity to normal parenchyma and not adjacent to acinar atrophy, duct dilation, or fibrosis were included to avoid bias in the analysis, as is in line with the revised recommendations of Fisher et al. from 2016 (21). Cells were identified as positive based on typical morphology and at least 50% of the cell membrane, cytoplasm, or nucleus stained positively. Independent cell counting was performed manually by two investigators (TKB, KS) to control for the inter-observer variability. Positive cells located in five manually annotated focal infiltrates were counted using the Counter Tool on a ×40-objective magnification for each MSG. However, in MSG possessing <5 focal infiltrates the positively stained cell populations in all infiltrates present were examined. To further minimize discrepancies, the same focal infiltrates were investigated for every staining performed in each pSS patient, whenever feasible (**Supplementary Table 2**). To exclude differences attributable to the severity of lesions, counting was expressed as the number of positive cells/total number of infiltrating mononuclear cells for each focal infiltrate, and the percentage of each immune cell population was deduced (**Table 2**).

**Table 2:** Composition of key immune cells in the focal infiltrates of patients included in the immunohistochemical analysis, expressed as the mean/median percentage of total infiltrating mononuclear cells.

Type of mononuclear cell	pSS patients				Statistical significance (*p*-value)		
	Inflammatory severity index				Statistical comparison		
	Total, n = 30 (%)	S1, n = 11 (%)	S2, n = 9 (%)	S3, n = 10 (%)	S1 *vs*. S2	S2 *vs*. S3	S1 *vs*. S3
**Mean CD4^+^ Th cells**	29.6 ± 1.71	33.09 ± 2.29	23.11 ± 2.56	31.50 ± 3.23	0.009 ^d^	0.061 ^d^	0.688 ^d^
**Mean CD8^+^ Tc cells**	21.7 ± 1.88	23.73 ± 2.97	21.00 ± 3.19	20.20 ± 3.81	0.541 ^d^	0.876 ^d^	0.470 ^d^
**Mean FoxP3^+^ Tregs**	0.70 ± 0.16	0.82 ± 0.26	0.56 ± 0.34	0.70 ± 0.26	0.456 ^c^	0.604 ^c^	0.809 ^c^
**Median FoxP3^+^ Tregs**	0.00 [0.00–1.00]	1.00 [0.00–2.00]	0.00 [0.00–1.00]	0.50 [0.00–11.25]			
**Mean CD74^+^ APCs**	35.7 ± 2.60	32.36 ± 4.31	36.33 ± 4.73	38.70 ± 4.71	0.543 ^d^	0.604 ^c^	0.349 ^c^
**Median CD74^+^ APCs**				46.0 [31.75–48.50]			
**Mean CD68^+^ macrophages**	1.63 ± 0.41	2.00 ± 0.94	1.11 ± 0.56	1.70 ± 0.50	0.370 ^c^	0.356 ^c^	0.756 ^c^
**Median CD68^+^ macrophages**	1.00 [0.00–2.25]	1.00 [0.00–2.00]	0.00 [0.00–2.50]				
**Mean CD20^+^ B cells**	23.5 ± 2.79	16.64 ± 3.16	17.33 ± 2.65	36.50 ± 5.38	0.871 ^d^	0.004 ^c^	0.004 ^d^
**Median CD20^+^ B cells**	20.0 [12.00–30.50]						
**Mean CD138^+^ plasma cells**	12.1 ± 1.91	14.45 ± 3.52	14.67 ± 3.46	7.10 ± 2.50	0.603 ^c^	0.053 ^c^	0.072 ^c^
**Median CD138^+^ plasma cells**	8.00 [5.00–19.25]	8.00 [5.00–24.00]	13.0 [7.00–21.50]	4.00 [1.00–13.00]			

c Mann–Whitney U test.

d Student's T test. Significant values (p <0.05) underlined.

S1, Mild stage; S2, Moderate stage; S3, Severe stage along the Inflammatory severity index; APCs, Antigen-presenting cells. Mean (%) values presented with standard error of mean (SEM) when data was normally distributed, i.e. Shapiro–Wilk test p >0.05. Median (%) [IQR] was used when the data followed a non-normal distribution.

### Statistical Analysis

For the statistical analysis and comparison between groups, Chi-square and Fisher’s Exact Tests were applied primarily. Differences in means were investigated with Student’s T test and the accuracy of the results were presented using standard error mean (SEM). Continuous variables were explored using Shapiro–Wilk test and considered normally distributed at *p >*0.05. Mann–Whitney U test was carried out for non-parametric data and Pearson correlation test was used to determine the strength and direction of the linear regression. A *p*-value <0.05 was considered statistically significant. Analyses were performed using the IBM SPSS Statistics v. 20.0.0 software and statistical figures were generated using Prism 9 (GraphPad).

## Results

### Inflammatory Disease Severity in the MSG of pSS Patients Influence Adipose Tissue Replacement, Hyposalivation, and Autoantibodies

**Figure 2:**
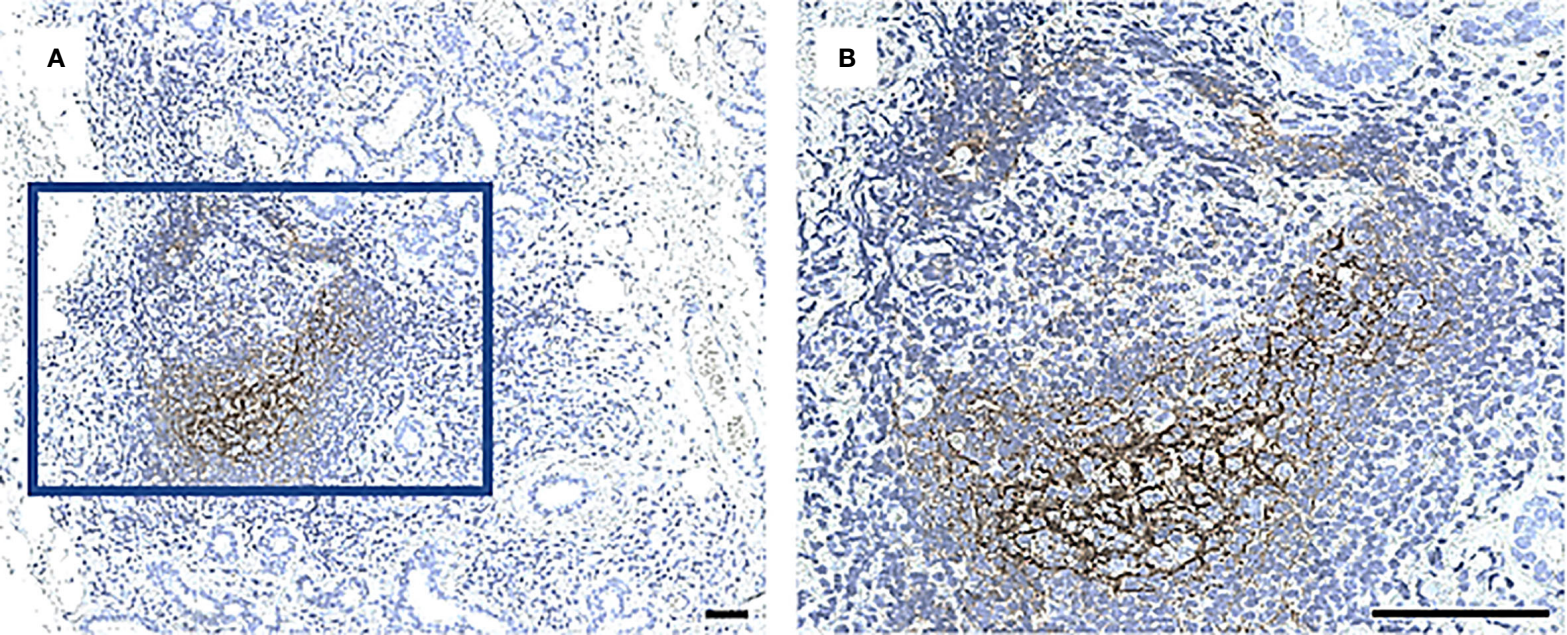
A CD21+ network of follicular DCs in GC in a MSG of a patient with pSS. Staining with CD21 antibody performed in all biopsies to identify a characteristic fDC-network formed in the light zone of GC. Image **(B)** is a magnification of **(A)**. Scale bar = 0.5 mm.

In this study, atrophic and adipose regions (**Figure 1**) were assessed in all control subjects, and in patients who were classified based on the glandular inflammation grade and upon the confirmation of GC (**Figure 2**) into the S1 (n = 44), S2 (n = 24) and S3 (n = 17). The mean glandular atrophy appeared consistent across all inflammatory severity stages, and between pSS patients compared to non-SS controls. Adipose tissue within the glandular parenchyma was 3.2% higher in MSG of patients compared to non-SS controls, although not significantly, and indicated a decreasing trend with greater inflammatory severity. Moreover, the mean age of diagnosis was 52.4 (± 1.22) for the pSS patients and 54.7 (± 1.41) for the non-SS sicca subjects, whereupon a positive correlation between the age and parenchymal adipose infiltration in both the patient (*r* = 0.458, *p <*0.0001) and non-SS sicca controls biopsies (*r* = 0.515, *p <*0.0001) was discovered. Conversely, age could not be associated with atrophy within the pSS patients, nor non-SS control biopsies. Furthermore, no association was observed between levels of atrophy and adipose infiltration in the MSG of the two groups. However, when the Chi-square test was applied to determine a relationship between mild and marked levels, it revealed that non-SS subjects with mild atrophy in their MSG exhibited mild adipose infiltration, while non-SS individuals with marked atrophy also displayed enhanced adipose deposition (*p <*0.01). The reciprocal pattern was not identified in the pSS patients.

The mean lacrimal flow of both eyes in the patients was measured and inversely correlated with their age (*r* = −0.268, *p <*0.05) and their focus score (*r* = −0.240, *p <*0.05). The percentage of patients with a positive Schirmer’s test increased with higher disease severity, where the incidence of patients with a positive test almost doubled in S3 compared to S1. Additionally, hyposalivation (≤1.50 ml/15 min) was considerably more common in S3 patients relative to those in the S2 group (*p <*0.05). Of note, patients in S2 measured a higher mean salivary flow relative to S1 and S3 patients combined (*p <*0.05).

Relatively to S1, a significantly higher prevalence of patients in S3 were seropositive for anti-Ro/SSA (*p <*0.05) and RF (*p <*0.05), and the percentage of patients with the respective autoantibodies increased with higher inflammatory disease severity. Meanwhile, anti-La/SSB positivity was less common in S1 compared to both S2 (*p <*0.01) and S3 (*p <*0.05) (**Table 1**). No correlations were observed between the aforementioned clinical parameters and the immunological patterns examined below (data not shown).

### Lower Numbers of T Cell Subsets Detected in the Target Organ of pSS Patients Reflecting High Inflammatory Disease Severity

**Figure 3:**
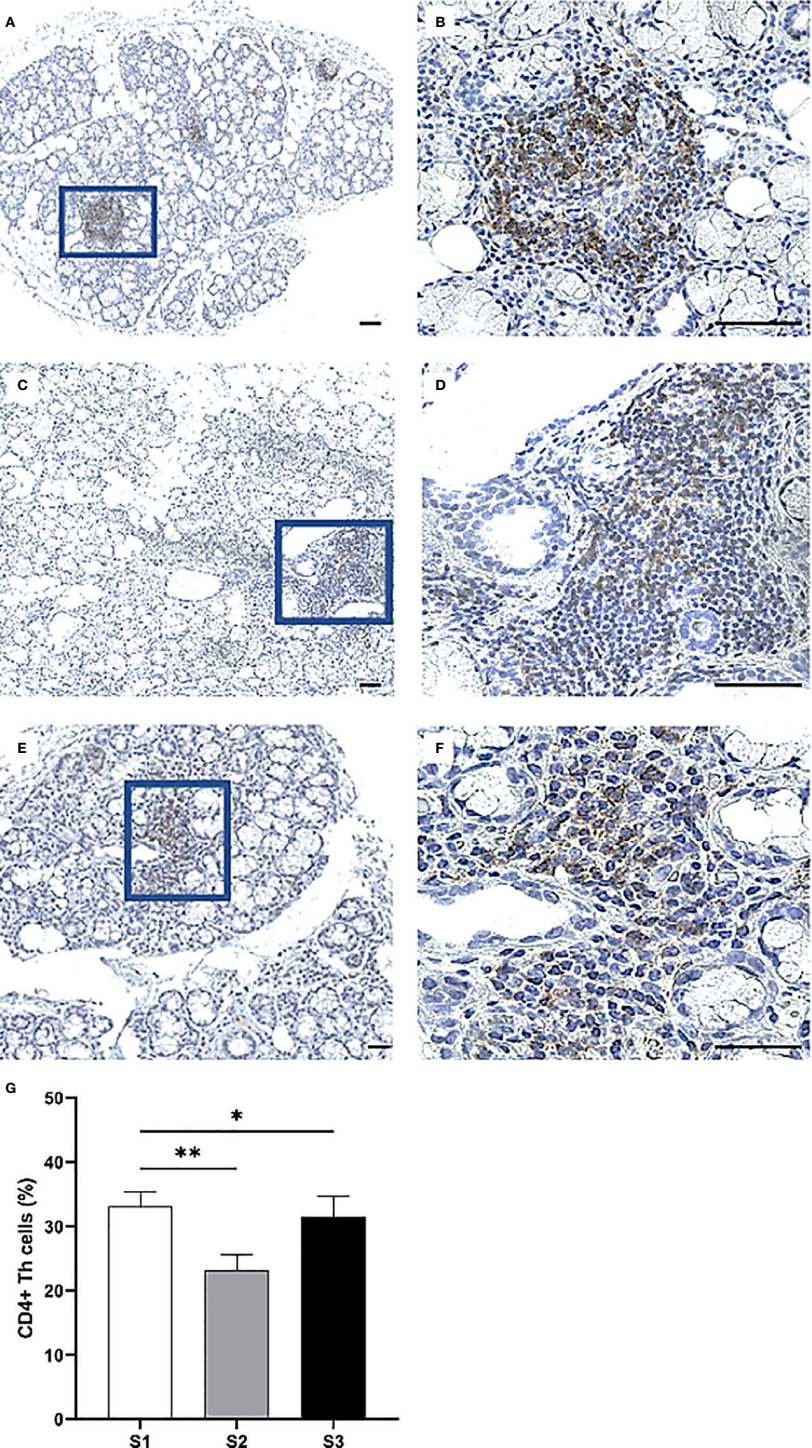
Immunohistochemical detection and quantification of CD4^+^ Th cells in the MSG of pSS patients. CD4^+^ Th cells were most frequently observed in the focal infiltrates of MSG across all inflammatory severity stages **(A–F)** and dropped significantly in S2 compared to S1 (*p* = 0.009), in addition to S2 compared to S1 and S3 combined (*p* = 0.011) as determined by Student's T test **(G)**. Immunohistochemical staining displaying the distribution of a specific immune cell in three representative examples of focal infiltrates in the MSG as classified into the respective inflammatory severity stages; S1 **(A, B)**, S2 **(C, D)**, and S3 **(E, F)**. Image **(B)** is a magnification of **(A)**, image **(D)** is a magnification of **(C)**, image **(F)** is a magnification of **(E)**. Scale bar = 0.5 mm. Bars **(G)** indicate the mean percentage of cell-type number/total infiltrating mononuclear cells in each stage and error bars represent ± SEM. Statistical significance is notated: * = p < 0.05, ** = p ≤ 0.01. The Student's T test was applied.

**Figure 4:**
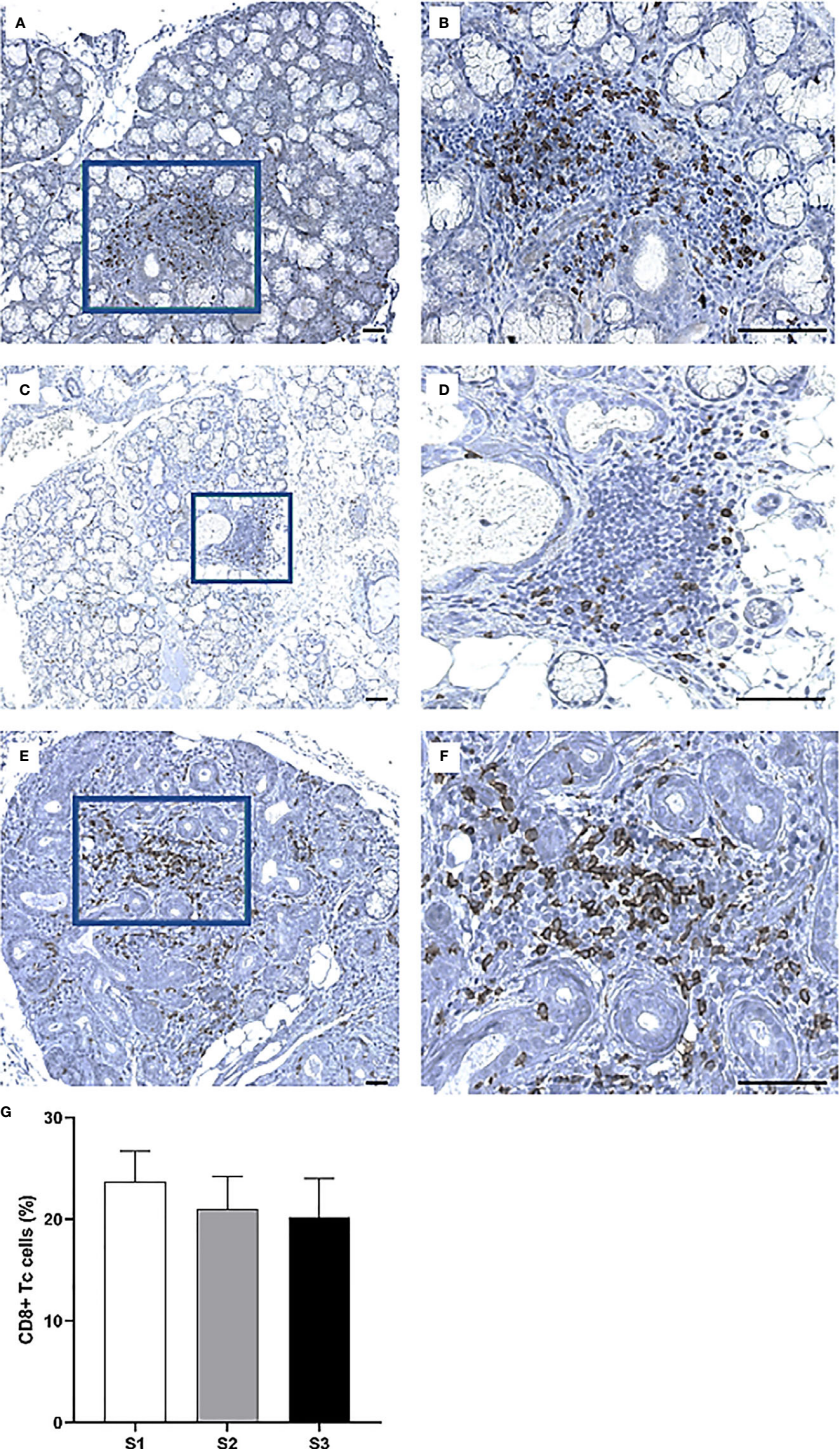
Visualization of CD8^+^ Tc cells in the MSG of pSS patients. CD8^+^ cells were observed in both periductal foci and within the epithelial lining of salivary ducts, where such ducts mostly appeared to be non-dilated **(A–F)**. Interestingly, the incidence of CD8^+^ cells fluctuated largely between patients regardless of lesion severity **(G)**. Immunohistochemical staining displaying the distribution of a specific immune cell in three representative examples of focal infiltrates in the MSG as classified into the respective inflammatory severity stages; S1 **(A, B)**, S2 **(C, D)**, and S3 **(E, F)**. Image **(B)** is a magnification of **(A)**, image **(D)** is a magnification of **(C)**, image **(F)** is a magnification of **(E)**. Scale bar = 0.5 mm. Bars **(G)** indicate the mean percentage of cell-type number/total infiltrating mononuclear cells in each stage and error bars represent ± SEM. The Student's T test was applied.

**Figure 5:**
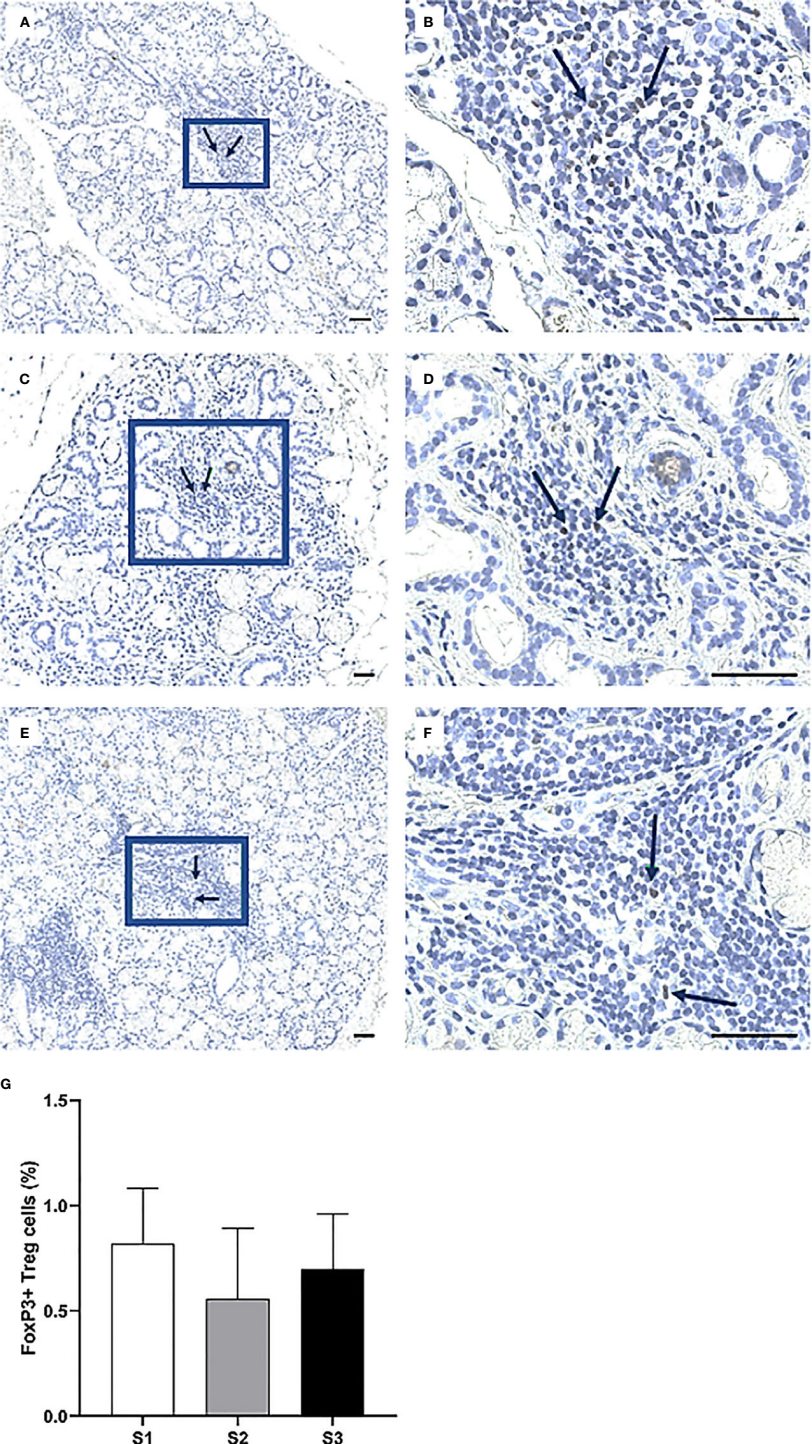
Identification of FoxP3^+^ cells in the MSG of patients with pSS. Here, some of the detected FoxP3^+^ Tregs are indicated by arrows **(A–F)**. FoxP3^+^ cells were scarce or absent in the patients, particularly in the S2 group. Although the alternations in FoxP3^+^ cell-incidence in accord with inflammatory disease severity resembled that of CD4^+^ Th cells, no significant differences were noted for this subpopulation **(G)**. Immunohistochemical staining displaying the distribution of a specific immune cell in three representative examples of focal infiltrates in the MSG as classified into the respective inflammatory severity stages; S1 **(A, B)**, S2 **(C, D)**, and S3 **(E, F)**. Image **(B)** is a magnification of **(A)**, image **(D)** is a magnification of **(C)**, image **(F)** is a magnification of **(E)**. Scale bar = 0.5 mm. Bars **(G)** indicate the mean percentage of cell-type number/total infiltrating mononuclear cells in each stage and error bars represent ± SEM. The Student's T test was applied.

Immunohistochemistry was performed to quantify immune cells in the MSG of patients in the severity stages S1 (n = 11), S2 (n = 9), and S3 (n = 10). Three sub-populations of T cells (CD4^+^ Th cells, CD8^+^ Tc cells, and FoxP3^+^ Tregs) were investigated. CD4^+^ Th cells were prominent in all patients from each stage and especially numerous in focal infiltrates of the S1 group (**Figures 3A–F**). A substantial difference in the number of positive cells was observed between S1 and S2, where the mean value of counted cells was 10% higher in S1 patients compared to S2 (*p <*0.01). The number of CD4^+^ Th cells increased from S2 to S3, although not significantly (**Figure 3G**). Meanwhile, CD8^+^ Tc cells were found mostly in the focal infiltrates but were less densely packed, and in some patients these cells were frequently observed within the excretory ducts close to the infiltrates (**Figures 4A–F**). The CD8^+^ cells constituted approximately 20% of the lymphocytic foci in all severity stages, and there appeared to be a slight decrease with higher inflammatory severity (**Figure 4G**). Moreover, the FoxP3^+^ Tregs were scarce or absent in the majority of MSG (**Figures 5A–F**), yet the incidence of the cells was found to follow a similar trend as that of the CD4^+^ Th cells, with the highest mean percentage of cells detected in S1 and the lowest number observed in S2 patients (**Figure 5G**).

### Increased Infiltration of APCs at the Site of Inflammation in pSS Patients With Higher Inflammatory Disease Severity

**Figure 6:**
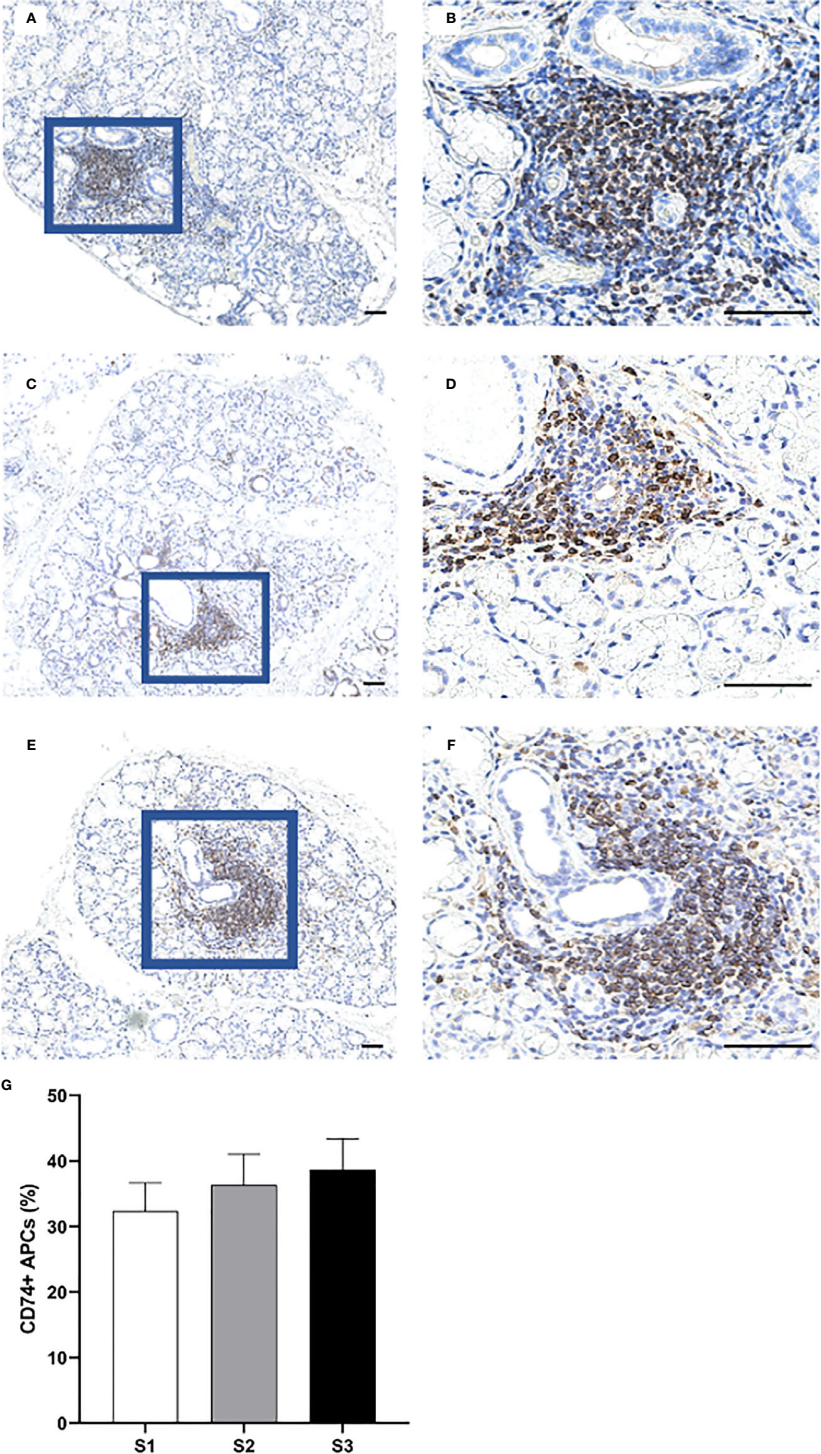
Distribution of CD74^+^ cells in the MSG of patients with pSS. CD74^+^ APCs were found to predominantly accumulate within the inflammatory lesions **(A–F)**, and steadily increased in parallel with higher lesion severity **(G)**. Immunohistochemical staining displaying the distribution of a specific immune cell in three representative examples of focal infiltrates in the MSG as classified into the respective inflammatory severity stages; S1 **(A, B)**, S2 **(C, D)**, and S3 **(E, F)**. Image **(B)** is a magnification of **(A)**, image **(D)** is a magnification of **(C)**, image **(F)** is a magnification of **(E)**. Scale bar = 0.5 mm. Bars **(G)** indicate the mean percentage of cell-type number/total infiltrating mononuclear cells in each stage and error bars represent ± SEM. The Student's T test and Mann–Whitney U test were applied.

**Figure 7:**
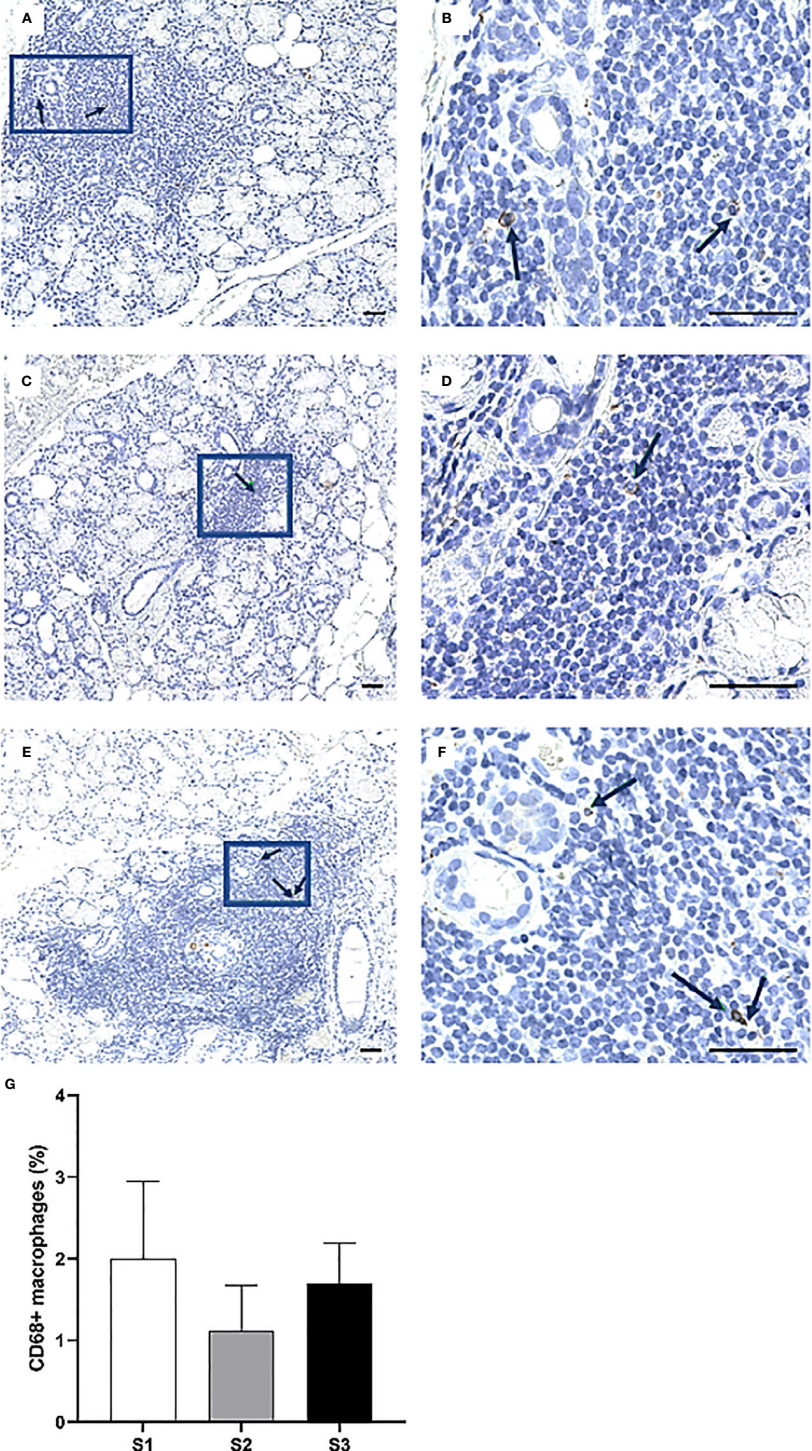
Presence of CD68^+^ cells in the MSG of patients with pSS. The black arrows indicate detected CD68^+^ macrophages. The cells appeared co-localized and morphologically larger inside the heavier focal infiltrates of S3 patients **(A–F)**, yet the highest percentage of cells was detected in S1 lesions. Immunohistochemical staining displaying the distribution of a specific immune cell in three representative examples of focal infiltrates in the MSG as classified into the respective inflammatory severity stages; S1 **(A, B)**, S2 **(C, D)**, and S3 **(E, F)**. Image **(B)** is a magnification of **(A)**, image **(D)** is a magnification of **(C)**, image **(F)** is a magnification of **(E)**. Scale bar = 0.5 mm. Bars **(G)** indicate the mean percentage of cell-type number/total infiltrating mononuclear cells in each stage and error bars represent ± SEM. The Mann–Whitney U test was applied.

Overall, the CD74^+^ expressing APCs represented the most abundant cell population in the focal infiltrates (**Figures 6A–F**) and increased in parallel with greater inflammatory disease severity, where S3 patients displayed a mean amount of approximately 40% positive cells (**Figure 6G**). Additionally, CD68^+^ macrophages were mostly found to be scattered and localized either interstitially or on the periphery of the infiltrates at the site of inflammation. Focal infiltrates were more enriched with this subgroup of APCs as compared to the GC (**Figures 7A–F**). This observation fits well with the small change in incidence of CD68^+^ macrophages discovered with respect to increasing inflammatory severity, as the highest occurrence rate was encountered in S1 and the smallest was seen in the S3 group (**Figure 7G**). Nevertheless, the total number of APCs occupying the infiltrates, with macrophages included, was highly elevated in S3 patients.

### Accumulation of CD20^+^ B Cells in MSGs of pSS Patients Displaying High Inflammatory Disease Severity and a Decline in CD138^+^ Plasma Cells

**Figure 8:**
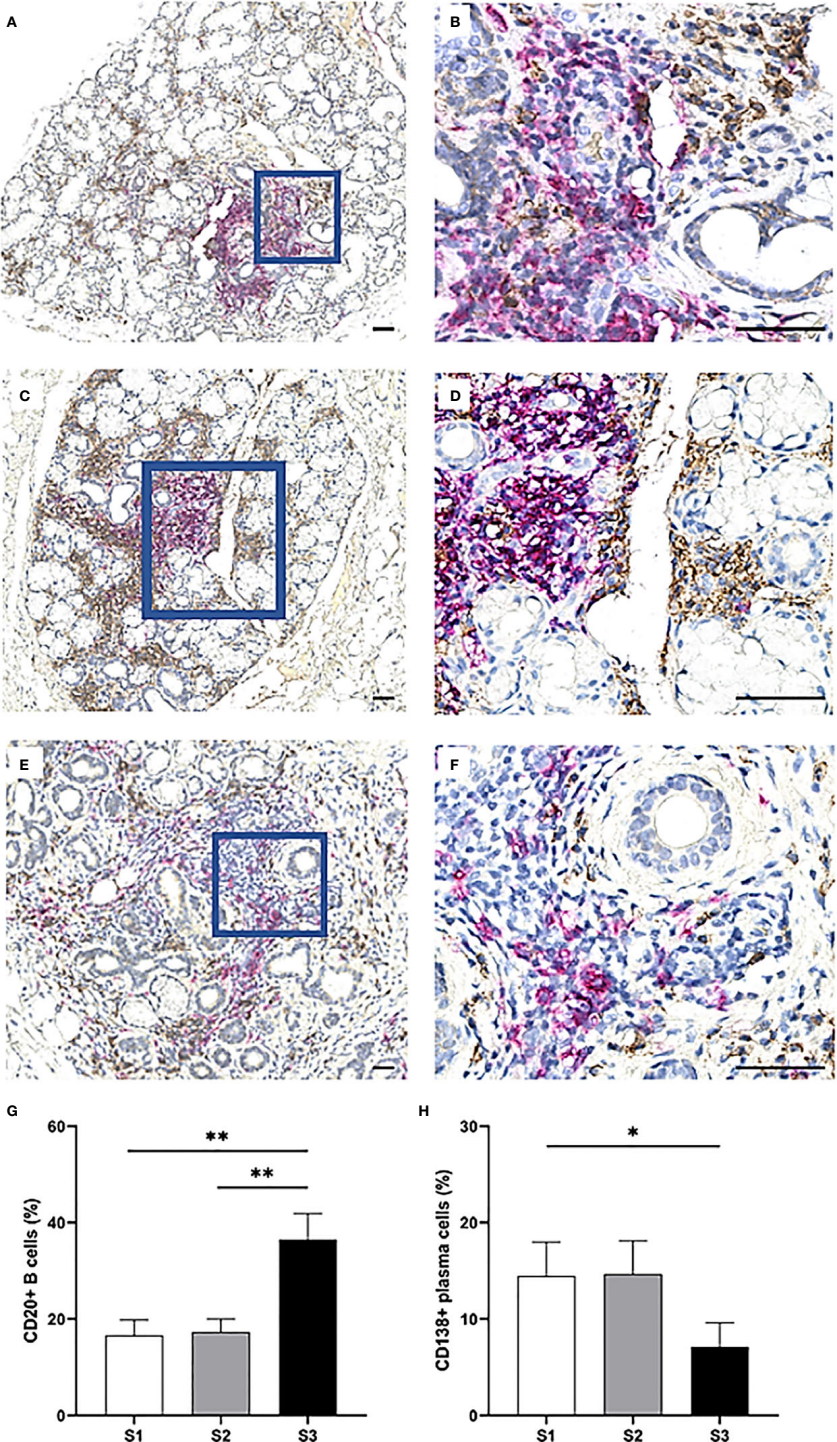
Distribution of CD20^+^ B cells and CD138^+^ plasma cells in the MSG of pSS patients. CD20^+^ B cells (magenta) were mostly observed in large numbers within large focal infiltrates and GC, in contrast to CD138^+^ plasma cells (brown) which were localized throughout the glandular tissue. However, the double staining revealed that both cell-types were commonly found adjacent to adipocytes **(A–F)**, unlike the other mononuclear cells in this study. CD20^+^ B cells significantly increased from S1 to S3 (*p* = 0.004) as tested by the Student's T test, from S2 to S3 (*p* = 0.004) as tested by the Mann–Whitney U test and were elevated in S3 compared to S1 and S2 combined (*p <* 0.001) as tested by the Student's T test. On the other hand, a higher incidence of CD138^+^ plasma cells in S1 and S2 lesions together was discovered compared to S3 using the Mann–Whitney U test (*p* = 0.028). Immunohistochemical staining displaying the distribution of a specific immune cell in three representative examples of focal infiltrates in the MSG as classified into the respective inflammatory severity stages; S1 **(A, B)**, S2 **(C, D)**, and S3 **(E, F)**. Image **(B)** is a magnification of **(A)**, image **(D)** is a magnification of **(C)**, image **(F)** is a magnification of **(E)**. Scale bar = 0.5 mm. Bars **(G, H)** indicate the mean percentage of cell-type number/total infiltrating mononuclear cells in each stage and error bars represent ± SEM. Statistical significance is notated: * = p < 0.05, ** = p ≤0.01. The Student's T test and Mann–Whitney U test were applied.

The CD20^+^ B cells heavily infiltrated the focal infiltrates of patients with GC, while the CD138^+^ plasma cells appeared to be rather condensed into clusters interstitially. Both cell types were commonly located in various regions of adipose deposition (**Figures 8A–F**). Strikingly, the CD20^+^ B cells exhibited the greatest variation in number amongst the three severity stages and were significantly up-regulated in MSG of S3 patients as compared to S1 (*p <*0.01), S2 (*p <*0.01) and both groups combined (*p <*0.01) (**Figures 8G**). On the other hand, the frequency of CD138^+^ plasma cells remained relatively consistent in both S1 and S2, yet diminished in S3, thereby giving rise to a considerable difference when comparing S3 with the S1 and S2 groups collectively (*p <*0.01) (**Figure 8H**).

## Discussion and Conclusion

The histopathology of salivary glands in pSS is of particular interest as several clinical, biological, and immunological aberrations have been directly attributed to changes that occur in the patients’ glandular tissue (3, 4, 22, 23). At the same time, only a few published studies have concerned the extent of atrophy and adipose tissue in the disease target organ, although both features represent important degenerative changes where functional acinar cells are damaged or replaced (8). To the best of our knowledge, we are the first to present data that mild and marked atrophy levels in the salivary gland parenchyma of non-SS sicca controls significantly coincided with the respective levels of adipose infiltration, and that a similar parallel could not be drawn in the pSS patients (*p <*0.01). Common to both groups was a positive correlation between the age of diagnosis and adipose accumulation in the salivary glands, as has been demonstrated in patients previously (11, 24). On the other hand, adipocytes have been reported to localize inside favourable microenvironments, including interleukin (IL)-6 and IL-17 rich milieus that associate with the glandular inflammatory activity in pSS (14, 25). Additionally, the release of adiponectin by salivary gland epithelial cells could be in favour of the infiltrating adipocytes (26). Therefore, multiple biological and pathological mechanisms may collectively support the proliferation or presence of these cells within the salivary glands, also considering that a slightly higher mean adipose accumulation was detected in pSS patients as compared to non-SS individuals.

In this study, pSS patients with GC generally experienced reduced mean salivary flow (4, 27), meanwhile, it appears that salivary secretion largely but not consistently correlated with the FS (27). Here, we demonstrated a significantly reduced salivary flow in patients with mild and severe lesions collectively, compared to patients with moderate lesions. As expected, seropositivity to ANA, anti-Ro/SSA, and RF were significantly more common in the S3 group of patients (3, 4). Interestingly, the difference in detected anti-La/SSB between patients with moderate lesions and severe lesions was insignificantly small (**Table 1**).

Inflammation grade has been reported to influence the shift of infiltrating immune cell populations within the MSG, however, the changes are not extensively described and there appears to be a lack of consistency in the available data. This could be explained by methodological variations ascribed to evaluating the cell occurrence (e.g., cells per randomly selected tissue-area, number of cells in infiltrates), the grading system for inflammation (e.g., focus score, Tarpley score, Chisholm score), and the patient stratification (e.g., based on different histological or clinical features). In the current study, the stained immune cells were quantified within the focal infiltrates, which are hallmark features in the pSS biopsies that can be used to describe inflammatory disease severity and should follow certain criteria with respect to the infiltrate’s cellular quantity and its surrounding tissue, in agreement with the recommendations by Fisher et al. (21). To allow for a broader understanding of the early biological changes that contribute to disease initiation, pSS patients with FS 0 and FS <1 were enrolled, as has been recommended in previous studies (1, 28). Since several reports have emphasized differences in disease activity status and cytokine profiles between GC^+^ and GC^−^ pSS patients, they were separated by using the CD21 marker in addition to the H&E evaluation for detection of follicular dendritic cell networks present within the ectopic GC structures (19, 29).

The focal infiltrates were found to mainly comprise CD74^+^ cells, CD4^+^, and CD20^+^ cells. The CD20^+^ cells clearly outnumbered the other CD74-expressing APCs; DCs, B cells, and macrophages. The anti-human CD74 recognizes the MHC class II invariant chain which is crucial for the formation, transportation, and function of the MHC class II molecule following antigen-binding. Additionally, the CD74 pathway plays a crucial role in B cell survival and has been described as complementary to that of the B cell receptor (BCR) and the B cell activating factor receptor (BAFFR) (30). Interestingly, CD20^+^ B cells comprised approximately half of the CD74^+^ APCs localized within the mild and moderate inflammatory lesions, respectively, and accounted for the vast majority of CD74^+^ APCs in the severe lesions. Moreover, the number of these B cells was further increased in severe lesions.

Besides recognizing antigens presented by APCs through the class II MHC molecules, CD4^+^ Th cells express the CD74 ligand macrophage migration inhibitory factor (MIF) and are key players in promoting B cell survival signaling pathways, for example through the activation of nuclear factor-kappa-B (NF-κB) pathway, resulting in the transcription of anti-apoptotic genes in these cells (30). In line with previous reports, it was observed that CD4^+^ Th cells dominated over the CD8^+^ Tc cells, particularly at earlier timepoints of the inflammatory course (15, 28, 31). One of the most extensively studied CD4^+^ Th cells that gain access into the GC are the T follicular helper (Tfh) cells which are up-regulated in the salivary glands of pSS patients (32, 33), and have been directly linked to support differentiation of antigen-specific B cells into memory B cells and plasma cells at these sites (34). In the present study, CD4^+^ Th cells were found to largely accumulate in S3 patients comparatively to patients with a higher focus score and absence of GC. However, as a significant difference was not detected, further exploration to uncover the suggested shift in the CD4^+^ Th cell subpopulations could provide a better understanding of the development of these intricate structures in the target tissue.

In contrast to the B cells from which they differentiate, CD138^+^ plasma cells in the focal infiltrates significantly declined in the MSG of S3 patients. According to a study from 2011 by our group where MSG biopsies from pSS patients were classified into FS = 1 and FS ≥2 irrespective of GC, there was an insignificant difference in detected CD138^+^ plasma cells in the focal infiltrates between the two groups, meanwhile, a significant increase in interstitial CD138^+^ plasma cells of biopsies with FS ≥2 was reported when compared to the other FS = 1 group (20). In this context, the plasma cells could possibly be elevated in the MSG of S3 patients but rather accumulate interstitially and form clusters or “infiltrates” of their own. This is further supported by the GC and over-expressed plasma cell survival factors, including CXCL12 and IL-6, which are released by epithelial cells, mononuclear cells, and adipocytes in the salivary glands of pSS patients (14, 20). Arguably, this may to some extent justify the preferred localization of plasma cells adjacent to adipocytes in the interstitium as demonstrated in the current study (**Figures 8A–F**), and in accordance with previous reports (14, 20). Nevertheless, further data is required to elucidate this concept.

Meanwhile, CD68^+^ macrophages and FoxP3^+^ Tregs constituted the least common of all cell types investigated within the focal infiltrates. As might be anticipated given that both cells express the CD4 co-receptor, the percentages of both CD68^+^ macrophages and FoxP3^+^ Tregs detected within the focal infiltrates appeared to fluctuate insignificantly over the course of the inflammatory progression in a relatively similar fashion to CD4^+^ Th cells. Although the highest incidence of CD68^+^ macrophages were immunolocalized in the severe lesions (35), the detected rise in cells associated with the mild lesions could indicate an active role in orchestrating tissue injury as part of the initial, innate immune response which could, in turn, possibly be linked to the following increase in atrophy in the S2 group (**Table 1**). Similar to the CD68^+^ macrophages, the FoxP3^+^ cells were reduced in the moderate lesions, although differences were insignificant. On the other hand, these cells have been found to increase with higher salivary gland infiltration grade (36). Interestingly, we did identify the highest cell incidence in the S1 patients’ mild inflammatory lesions, probably reflecting the immune system’s attempt to efficiently control local immune responses in these patients following disease progression.

In summary, our findings provide novel insights into the pSS disease pathogenesis, specifically by investigating the significance of the salivary gland degradation which is usually coupled with adipose tissue occurrence and immune cell alternations in the focal infiltrates, and how they associate with clinical outcomes. This could add valuable knowledge to the current understanding of the disease pathogenesis and be directly transferred to the optimization of available diagnostic strategies applied for pSS patients in distinct inflammatory disease severity stages. Further assessments of atrophy and adipose tissue in the context of immunopathological cell aberrations in the salivary glands should be encouraged, for example by imaging modalities and cytometry platforms.

## Data Availability Statement

The original contributions presented in the study are included in the article/**Supplementary Material**. Further inquiries can be directed to the corresponding author.

## Ethics Statement 

The studies involving human participants were reviewed and approved by Regional committee of Ethics, Western-Norway (2009/686). The patients/participants provided their written informed consent to participate in this study.

## Author Contributions

KS and SA conceived and designed the study. TB, KS, and LA drafted the manuscript. SA, RJ, JB, and SF critically evaluated the manuscript. TB conducted data acquisition, processing, and analysis. LA and TB carried out data presentation. KS, SA, LA, and TB contributed to the interpretation of data, and critically revising the results. TB and SF performed the immunohistochemical procedures. RJ, JB, and SA were involved in the clinical data acquisition. RJ provided the biopsy material. All authors contributed to the article and approved the submitted version.

## Funding

This study was funded and supported by the Student Research Programme and the Depatment of Clinical Medicine at the Faculty of Medicine, University of Bergen, and Faculty of Medicine, University of Bergen, and the Western Norway Regional Health Authority.

## Conflict of Interest

The authors declare that the research was conducted in the absence of any commercial or financial relationships that could be construed as a potential conflict of interest.
