# Supplementary material for: Inflammatory Stratification in Primary Sjögren’s Syndrome Reveals Novel Immune Cell Alterations in Patients’ Minor Salivary Glands
Source: Front Immunol. 2021 Jul 12;12:701581. doi: 10.3389/fimmu.2021.701581 (PMC8311440; doi:10.3389/fimmu.2021.701581)
Supplement: Supplementary file 1 [file Table_1.docx]

**Supplementary Table 1** Mean/median values and number (%) of patients from the immunohistochemical analysis positive for the clinical

and histopathological characteristics

|  | **pSS patients** | | | | **Statistical significance (*p*-value)** | | |
| --- | --- | --- | --- | --- | --- | --- | --- |
|  |  | Inflammatory severity index | | | Statistical comparison | | |
|  | **Total, n=30 (%)** | **S1, n=11 (%)** | **S2, n=9 (%)** | **S3, n=10 (%)** | **S1 vs. S2** | **S2 vs. S3** | **S1 vs. S3** |
| **Female** | 30/30 (100) | 11/11 (100) | 9/9 (100) | 10/10 (100) |  |  |  |
| **Mean age (years)** | 50.2 ± 2.15 | 50.2 ± 3.17 | 47.9 ± 4.69 | 52.3 ± 3.73 |  |  |  |
| **ANA+^*^** | 21/26 (80.8) | 9/11 (81.8) | 5/6 (83.3) | 7/9 (77.8) | 1.000 ^b^ | 1.000 ^b^ | 1.000 ^b^ |
| **Anti-Ro/SSA+^*^** | 17/30 (56.7) | 5/11 (45.5) | 4/9 (44.4) | 8/10 (80.0) | 1.000 ^b^ | 0.170 ^b^ | 0.119 ^a^ |
| **Anti-La/SSB+^*^** | 9/30 (30.0) | 2/11 (18.2) | 2/9 (22.2) | 5/10 (50.0) | 1.000 ^b^ | 0.350 ^b^ | 0.183 ^b^ |
| **RF+^*^** | 5/30 (16.7) | 1/11 (9.1) | 2/9 (22.2) | 2/10 (20.0) | 0.421 ^b^ | 1.000 ^b^ | 0.586 ^b^ |
| **Mean FS ^**^**  **Median FS^**^** | 2.18 ± 0.25  2.00 [1.00-  3.00] | 0.86 ± 0.07  1.00 [0.50-  1.00] | 2.67 ± 0.29  2.00 [2.00-  3.50] | 3.20 ± 0.44  3.00 [2.00-  4.25] |  |  |  |
| **GC^+^** | 10/30 (33.3) | 0 (0) | 0 (0) | 10/10 (100) |  |  |  |
| **Mean atrophy^***^ Median atrophy^***^** | 4.47 ± 0.49  3.50 [2.60-  5.75] | 4.48 ± 0.87 | 4.55 ± 1.16 | 4.38 ± 0.54 | 0.964 ^d^ | 0.892 ^d^ | 0.921 ^d^ |
| **Marked atrophy^***^** | 19/30 (63.3) | 7/11 (63.6) | 4/9 (44.4) | 8/10 (80.0) | 0.653 ^b^ | 0.170 ^b^ | 0.635 ^b^ |
| **Mean adipose tissue^***^ Median adipose tissue^***^** | 6.67 ± 2.07  1.47 [0.49-  9.53] | 4.61 ± 1.89  0.90 [0.61-  12.20] | 10.6 ± 5.02  2.81 [0.03-  21.72] | 5.40 ± 3.83  1.47 [0.62-  2.98] | 0.923 ^c^ | 0.780 ^c^ | 0.973 ^c^ |
| **Marked adipose tissue^***^** | 9/30 (30.0) | 3/11 (27.3) | 4/9 (44.4) | 2/10 (20.0) | 0.642 ^b^ | 0.350 ^b^ | 1.000 ^b^ |
| **Mean UWSF^****^ Median UWSF^****^** | 2.13 ± 0.60  1.20 [0.00-  2.50] | 1.94 ± 0.94  0.80 [0.10-  2.50] | 3.59 ± 1.45 | 0.91 ± 0.36 | 0.380 ^c^ | 0.096 ^d^ | 0.553 ^c^ |

| **Decreased UWSF (hyposalivation)**  ********* | 17/27 (63.0) | 7/11 (63.6) | 3/8 (37.5) | 7/8 (87.5) | 0.370 ^b^ | 0.119 ^b^ | 0.338 ^b^ |
| --- | --- | --- | --- | --- | --- | --- | --- |
| **Mean Schirmer’s test********  **Median Schirmer’s**  **test******** | 11.57 ± 2.23  8.00 [1.88-  18.75] | 12.8 ± 4.18  8.50 [1.50-  19.50] | 12.3 ± 4.87 | 9.00 ± 3.67  3.50 [2.00-  15.38] | 0.941 ^d^ | 0.674 ^c^ | 0.716 ^c^ |
| **Positive Schirmer’s**  **test********* | 16/30 (53.3) | 6/11 (54.5) | 4/9 (44.4) | 6/10 (60.0) | 1.000 ^b^ | 0.656 ^b^ | 1.000 ^b^ |

^a^Chi-square test; ^b^Fischer's exact test; ^c^Mann-Whitney U test; ^d^Student’s T test. (*p*<0.05).

S1: Mild stage, S2: Moderate stage, and S3: Severe stage along the Inflammatory severity index; ANA: Antinuclear antibody; RF:

Rheumatoid arthritis; FS: focus score; GC: germinal center-like structures; UWSF: unstimulated whole saliva flow. Mean values presented

with standard error of mean (SEM) when data was normally distributed, i.e. Shapiro-Wilk test *p*>0.05. Median [IQR] was used when the

data followed a non-normal distribution.

* ANA, anti-Ro/SSA, and anti-La/SSB autoantibodies were detected using ELISA. RF was detected using Waaler’s test.

** A re-evaluation of the FS was carried out in all MSG. Values are the mean of the number of focal infiltrates/4mm^2^ area

comprising of at least 50 mononuclear cells. When FS<1 the numeric value was set to 0.5.

*** The morphometric areas of atrophy and adipose tissue were quantified as a percentage of the total MSG parenchymal area,

where ≥ 3% implicates marked levels, and <3 % implicates mild levels (number of patients with mild levels not shown).

**** Values are in mL/15 minutes. 3 subjects missing.

***** A salivary flow of ≤1.5 mL/15 minutes denotes a positive sialometry test.

****** Values are in mm/5 minutes. Mean of lacrimal flow from both eyes.

******* A lacrimal flow of ≤ 5mm/5 minutes in at least one eye denotes a positive Schirmer’s test.

**Supplementary Table 2:** Overview of the quantifications in the immunohistochemical analysis, expressed as the mean/median number of

infiltrating mononuclear cells and the total cell number within the focal infiltrates

|  | **pSS patients** | | | | |
| --- | --- | --- | --- | --- | --- |
|  |  |  | Inflammatory severity index | | |
|  | **Cell counts** | **Total, n=30** | **S1, n=11** | **S2, n=9** | **S3, n=10** |
| **Mean focal infiltrates (n≤5)** |  | 4.10 ± 0.20 | 3.27 ± 0.27 | 4.11 ± 0.39 | 5.00 ± 0.00 |
| **Mean CD4^+^ Th cells** | Positive cells | 200.33 ± 25.31 | 145.09 ± 24.30 | 154.33 ± 26.60 | 302.50 ± 56.21 |
| **Median CD4^+^ Th cells** |  | 176.5 [104.00-287.50] |  |  |  |
|  | Total cells | 662.37 ± 63.45 | 436.18 ± 62.13 | 629.67 ± 92.29 | 940.60 ± 111.49 |
| **Mean CD8^+^ Tc cells** | Positive cells | 131.53 ± 23.94 | 98.82 ± 16.32 | 103.11 ± 17.71 | 193.10 ± 65.81 |
| **Median CD8^+^ Tc cells** |  | 87.00 [70.75-160.50] |  |  | 119.00 [77-257.75] |
|  |  |  |  |  |  |
|  | Total cells | 611.60 ± 61.98 | 449.82 ± 53.91 | 508.56 ± 75.58 | 882.30 ± 128.89 |
|  |  | 592.50 [368.25-757.50] |  |  |  |
| **Mean FoxP3^+^ Tregs** | Positive cells | 4.03 ± 0.77 | 3.73 ± 0.87 | 3.89 ± 2.06 | 4.50 ±1.18 |
| **Median FoxP3^+^ Tregs** |  | 4.00 [0.00-6.25] |  | 1.00 [0.00-7.00] |  |
|  | Total cells | 272.67 ± 42.80 | 210.55 ± 39.37 | 255.56 ± 96.78 | 356.40 ± 83.92 |
|  |  | 266.00 [0.00-425.50] |  |  |  |
| **Mean CD74^+^ APCs** | Positive cells | 252.13 ± 33.87 | 149.36 ± 30.24 | 212.33 ± 44.99 | 401.00 ± 66.65 |
| **Median CD74^+^ APCs** |  | 213.50 [103.50-341.25] |  |  |  |
|  | Total cells | 647.73 ± 65.16 | 421.64 ± 47.31 | 549.33 ± 74.73 | 985.00 ± 118.24 |
|  |  | 559.50 [405.75-867.25] |  |  |  |
| **Mean CD68^+^** | Positive cells | 9.47 ± 1.83 | 7.64 ± 2.48 | 6.22 ± 2.45 | 14.4 ± 3.98 |
| **macrophages** |  | 6.50 [1.75-16.00] |  | 3.00 [0.00-12.00] | 13 [2.50-23.00] |
| **Median CD68^+^** | Total cells | 479.90 ± 91.19 | 287.45 ± 55.80 | 376.11 ± 104.79 | 785.00 ± 227.72 |
| **macrophages** |  | 409.00 [134.25-691.00] |  |  |  |
| **Mean CD20^+^ B cells** | Positive cells | 136.23 ± 29.90 | 60.18 ± 17.05 | 80.56 ± 9.03 | 270.00 ± 72.22 |
| **Median CD20^+^ B cells** |  | 82.50 [48.75-161.00] | 36.00 [22.00-65.00] |  |  |

|  |  |  |  |  | 180.50 [131.25-  339.75] |
| --- | --- | --- | --- | --- | --- |
|  | Total cells | 485.40 ± 40.68 | 336.36 ± 39.87 | 500.44 ± 63.05 | 635.80 ± 75.01 |
| **Mean CD138^+^ plasma** | Positive cells | 42.90 ± 4.41 | 43.27 ± 7.20 | 53.00 ± 6.42 | 33.40 ± 8.44 |
| **cells** |  |  | 36.00 [25.00-66.00] |  |  |
| **Median CD138^+^ plasma** | Total cells | 413.33 ± 42.48 | 417.91 ± 89.42 | 427.33 ± 59.96 | 395.70 ± 68.84 |
| **cells** |  | 347 [282.25-533.75] | 342.00 [225.00- |  |  |
|  |  |  | 444.00] |  |  |

S1: Mild stage, S2: Moderate stage, and S3: Severe stage along the Inflammatory severity index; Mean values presented with standard error

of mean (SEM) when data was normally distributed, i.e. Shapiro-Wilk test *p*>0.05. Median [IQR] was used when the data followed a non-

normal distribution.
